# Photocatalytic removal of imidacloprid pesticide from wastewater using CdS QDs passivated by CQDs containing thiol groups

**DOI:** 10.1038/s41598-023-49972-8

**Published:** 2024-01-04

**Authors:** Homa Targhan, Aram Rezaei, Alireza Aliabadi, Ali Ramazani, Zhefei Zhao, Xinyi Shen, Huajun Zheng

**Affiliations:** 1 Nano Drug Delivery Research Center, Health Technology Institute, Kermanshah University of Medical Sciences, Kermanshah, Iran; 2 Pharmaceutical Sciences Research Center, Health Institute, School of Pharmacy, Kermanshah University of Medical Sciences, Kermanshah, Iran; 3 Department of Chemistry, University of Zanjan, Zanjan, 45371-38791 Iran; 4 Department of Applied Chemistry, Zhejiang University of Technology, Hangzhou, 310032 China

**Keywords:** Catalysis, Environmental chemistry, Photochemistry

## Abstract

Over the past decade, CdS QDs have become versatile semiconductors. Surface modification of CdS QDs has become an interesting case study, as it can eliminate surface defects and improve their photochemical properties. In this study, we report a new strategy of using carbon quantum dots containing a large number of thiol groups (CQDs-SH) as a passivating agent for the stabilization of CdS quantum dots (QDs). Various characterization techniques have clearly revealed that the CdS QDs have been successfully passivated by CQDs-SH. The photocatalytic performance of CQDs-SH/CdS QDs was investigated for the degradation of the insecticide imidacloprid from an aqueous solution. Parameters affecting the photodegradation process, including the light source, photocatalyst amount, initial concentration of the pollutant, radiation time, pH, oxidizing agent, and temperature, were investigated. Furthermore, the HPLC technique was applied to quantitatively analyze imidacloprid and its degradation products. The results of the HPLC analysis revealed that under simulated visible light at pH 9, imidacloprid scarcely existed after 90 min of irradiation (90.13% degradation). The LC–MS method was also used to detect the degradation products and investigate the mechanism of photodegradation of the pesticide. The results showed that the CQDs-SH/CdS QDs composite was a promising photocatalyst for the degradation of imidacloprid in wastewater.

## Introduction

It is well known that human activities such as industrialization and agricultural practices inevitably result in numerous environmental pollutants^1^. Among these substances, pesticides, which are used to protect plants from weeds, fungi, and insects, have become one of the greatest pollutants detected in freshwater (surface and groundwater) due to their widespread use in agriculture and improper methods of wastewater disposal^2^. According to United Nations reports, only 5% of all pesticides used in agriculture target pests, and less than 1% of the pesticides actually reach the crops, resulting in pesticide residues contaminating almost every part of our environment, including land, air, and particularly water^3,4^. Pesticide contamination can pose significant risks to the environment, from soil microorganisms to plants, insects, fish, birds, and human health. It is therefore not surprising that approximately 10,000 deaths and approximately 2 million poisonings occur from pesticides each year throughout the world^5,6^. On the other hand, the use of pesticides is inevitable in the interests of agriculture because yields could decrease by as much as a third without pesticides, and this, in turn, leads to an increase in the food price level by as much as 75%^7^. Imidacloprid, a potent neonicotinoid insecticide, represents one example of a pesticide used for seed treatment to effective control of the insects. Imidacloprid shows a high activity against the sucking and biting insects, therefore has become one of the most widely used insecticides. Experimental studies have been shown that imidacloprid is highly toxic and has good solubility and stability in water, therefore cause significant environmental concerns^8^.

Sunlight photolysis is a natural method for degrading persistent organic pollutants such as pesticides, which in turn reduces their accumulation in the environment. However, natural sunlight photodegradation typically leads to the formation of various intermediates and by-products, some of which can be more harmful than the original pesticide. Additionally, the direct photolysis rates of organic pollutants in natural aquatic systems under sunlight depend on factors such as light intensity, angle of incidence, and depth^9,10^. As a result, advanced oxidation processes (AOPs) have emerged as a new technology for degrading various organic contaminants in water, attracting a great deal of attention. AOPs rely on the production of strong oxidants, such as hydroxyl radicals, as their main mechanism of action. Among the different available AOPs, photocatalytic degradation using catalysts in combination with UV or visible light is considered a well-suited tool for treating various types of water pollutants^11–15^.

The size of particles plays a pivotal role in determining the performance of particulate photocatalysts. Nanoscale photocatalysts, renowned for their high surface area-to-volume ratio, generally exhibit superior activity when compared to bulk materials. Notably, nanoparticles of CeO_2_, TiO_2_, and ZnO with sizes ranging from 25 to 30 nm have emerged as highly desirable photocatalysts for various industrial applications due to their excellent photocatalytic activity and biocompatible nature^16,17^. However, traditional photocatalysts like TiO_2_ predominantly demonstrate effectiveness under UV light, while a substantial portion of solar radiation is present in the visible region. Consequently, there is a growing demand for nanoscale semiconductors possessing narrow bandgap energies in the range of 1.5–2.5 eV, which enables efficient absorption of sunlight. Such materials are sought after to enhance the utilization of solar energy for photocatalytic processes^18–20^.

Among the various technologies, quantum dots (QDs) with sized below 15 nm show great promise in capturing sunlight across a wide spectrum due to their high extinction coefficients and surface-to-volume ratios^21,22^. Of all the available semiconductors, CdS QDs have become increasingly important and have been studied for a wide range of applications due to their superior optical and electronic characteristics and great band gap^23^. However, several constraints, such as the tendency of CdS QDs to aggregate and photobleaching, which in turn leads to the deterioration of their optical properties, and the toxicity of bare core Cd QDs, greatly prevent their wide applications^24,25^. On the other hand, the environmental problems instigated by the tiny size and toxicity of QDs should not be underestimated. Studies indicate that sizes and surface properties of QDs can have a significant impact on the toxicity. Coating of QD’s core with passivating shell becomes one common way to reduce the toxicity and enhance biocompatibility and stability^18^. As a result, novel surface passivation approaches have been developed, such as coating with a thin layer of various shells, growing and embedding CdS QDs in matrices, and combining them with other semiconductors and carbon materials^26,27^. Thiol derivatives are usually employed for the passivation of CdS QDs, since the sulfur atoms are amenable to insertion within the crystalline phase of CdS QDs^28^. The passivation of CdS QDs has been carried out with numerous surfactants and organic compounds containing thiol groups, such as thioglycerol chains^29^, N-acetyl-L-cysteine^30^, mercaptopropionic acid^31^, thioglycolic acid^32^, glutathione^33^, mercaptosuccinic acid^34^, (3-mercaptopropyl)-trimethoxysilane^35^, and thiol-laced MOF UiO-66 (UiO-66-(SH)_2_)^36^. Consequently, the outstanding characteristics of CdS QDs passivated by thiol groups are well documented in the literature.

Carbon quantum dots (CQDs), a new class of nanocarbons, are considered potential candidates for photocatalytic applications due to their favorable properties, such as chemical and photo stability, solubility, low cost, low toxicity and efficient electrical conductivity^37–45^. Considering all of the beneficial properties of CQDs, an increasing body of research has attempted to apply them as an efficient component in the design of photocatalysts. Several studies have shown that the catalytic activity of CQDs can be improved through successful coupling with semiconductor photocatalysts (such as TiO_2_ and ZnO). Furthermore, CQDs are able to enhance the visible light photocatalytic activity of other semiconductor photocatalysts (such as Si, Ag_3_PO_4_, CdS, and Bi_2_WO_6_) through strengthening of visible light absorption region^15,46, 47^. Moreover, the existence of surface functional groups and a π-conjugated structure enables CQDs to be combined with other types of semiconductors. Therefore, CQDs have received great attention and have been widely applied in the photocatalysis field^48^.

In this project, our hypothesis is centered around synthesizing CQDs with thiol groups on their surface, enabling their utilization as passivating agents for QDs. By employing CQDs containing thiol groups as passivating agents, we aim to not only prevent the aggregation of CdS QDs but also enhance their photochemical stability and improve their photocatalytic performance. While previous research has been published on the synthesis of CdS/CQDs nanocomposites, none have investigated the use of CQDs containing thiol groups as passivating agents to stabilize CdS QDs and their application as photocatalysts. Hence, we present the passivation of CdS QDs using CQDs with a significant number of thiol groups. The deposition of a CQD layer to passivate the CdS QDs is anticipated to facilitate control over their size and stability during synthesis, ultimately resulting in enhanced photocatalytic efficiency. Consequently, we successfully fabricated a novel nanocomposite, CQDs-SH/CdS QDs, which exhibited superior photocatalytic activity in the decomposition of imidacloprid compared to CQDs-SH and CdS QDs.

## Experimental

### Materials and methods

All chemicals and solvents were purchased from Merck (Germany) or Fluka (Switzerland). The fourier-transform infrared spectroscopy (FT-IR) spectra of the samples were recorded with the KBr pellet method by PerkinElmer PE-1600-FTIR spectrometer. A SIGMA VP 500 (Zeiss) microscope equipped with an EDX measurement system was used to record field emission scanning electron microscope (FESEM) imaging and energy-dispersive X-ray spectroscopy (EDX) analysis. Transmission electron microscopy (TEM) images were obtained using a SIGMA VP 500 (Zeiss) microscope. X-ray diffraction (XRD) spectra were carried out using an X-ray diffractometer (PANalytical X'Pert PRO, Netherlands) with Cu Kα radiation (λ = 1.54 Å). An ESCALab MKII (Thermo Fisher Scientific, USA) spectrometer with Al Kα (1.4866 keV) as the X-ray source was used to record X-ray photoelectron spectroscopy (XPS). Zeta potential was measured using a Zetasizer Nano-ZS, Model ZEN3600 (Malvern Instruments Ltd, Malvern, UK).

### Synthesis of CQDs-SH

A convenient one-step approach, based upon customary method of CQDs synthesis was employed for the preparation of CQDs-SH. In a typical synthesis, and 100 mg citric acid, 300 mg 2-mercaptoacetic acid (2-MPA) and 321 mg 2-amino-5-mercapto-1,3,4-thiadiazole (AMT) were dispersed in 30 mL deionized water under ultrasound irradiation. Afterward, the resulted suspension was transferred into a Teflon autoclave. The autoclave was heated at 180 °C for 5 h and then allowed to down to room temperature. In order to completely remove any unreacted precursors, the resulted solution was then filtered through a dialysis membrane (100 Da) for 48 h. Ultimately, the CQDs-SH were obtained after freezer dryer for 24 h^40^.

Note: There are a number of potential risks (such as explosion and steam burns, etc.) associated with using an autoclave, therefore should be used only by experts who thoroughly understand the necessary precautions.

### Synthesis of CQDs-SH/CdS QDs

CQDs-SH/CdS QDs were prepared through simple aqueous chemical process. At the outset, CdCl_2_ 2.5 H_2_O (0.5 mmol, 0.114 g) and CQDs-SH (0.1 g) were dissolved in 50 ml deionized water. The 1 M NaOH solution was added drop-wise to the aqueous solution until pH 9 was reached. Under magnetic stirring, 1 mmol, 0.76 g of thiourea was then added to the reaction mixture. Afterwards, the reaction mixture was vigorously stirred at an oil bath temperature of 90 °C under N_2_ atmosphere for 2 h. The obtained suspension was centrifuged, washed with water three times and finally dried under vacuum at 50 °C overnight.

### Synthesis of CdS QDs

For synthesis of CdS QDs, CdCl_2_ 2.5 H_2_O (1 mmol, 0.228 g) and 3-mercaptopropionic acid (1.3 mmol, 0.113 mL) were dissolved in 50 ml deionized water. In the next step, 1 M NaOH solution was added until the solution showed pH 9. Under magnetic stirring, 1 mmol, 0.76 g of thiourea was then added to the reaction mixture. The obtained solution was heated with stirring at an oil bath temperature of 90 °C under nitrogen atmosphere. A color changing of solution (from colorless to yellow) indicates the formation of CdS QDs. By the addition of acetone, the synthesized CdS QDs were precipitated. After centrifugation, the CdS QDs were collected and finally dried at 60 °C.

### Experimental conditions for degradation of imidacloprid

In this study, the degradation experiments of imidacloprid were conducted in a reactor (50 mL volume) under simulated visible light irradiation intensity of 35 W LED lamp (Supplementary Fig. 1). The lamp was placed at a distance of 30 cm from the surface of the test solution. The pH adjustment of the imidacloprid solution was achieved using 0.1 M HCl and 0.1 M NaOH solutions. Six samples of imidacloprid solution (5 mL of 10 ppm) were prepared. After adding CQDs-SH/CdS QDs photocatalyst (1 g/L) to each imidacloprid solution, in order to reach adsorption–desorption equilibrium, the mixture solutions were kept in a dark chamber under magnetic stirring for 30 min. Each of the 6 resulting suspensions were exposed to simulated visible light for different irradiation times (the first suspension for 15 min and the subsequent suspensions 30, 45, 60, 75 and 90 min, respectively). In order to control the temperature of reaction a water-bath used. After separating the photocatalyst by centrifugation, the quantitative analysis of imidacloprid was performed using high performance liquid chromatography (HPLC). The experiment was repeated three times. Separations were carried out on a H5-ODS C18 column (25 cm × 4.6 mm ID, with 5 μm particle size) from Anachem Company (Luton, UK). The mobile phase (methanol/H_2_O:1/1) was flow at a rate of 1 mL/min. The degradation efficiency of imidacloprid pesticides (D%) was calculated using the following equation:

$${\text{D}}\left( \% \right) = \left( {\left( {{\text{C}}_{0} - {\text{C}}_{{\text{t}}} } \right)/{\text{C}}_{0} } \right) \times {1}00$$

Parameters C_0_ and C_t_ represent the initial concentration of pesticides and the pesticides concentration at different photodegradation times, respectively.

The various factors might have an influence on photodegradation of imidacloprid: light source, photodegradation process time, amount of the photocatalyst used, initial concentration of the pollutants, pH of substance solution, presence of an oxidizing agent and temperature. The effect of these parameters on the photodegradation process have thoroughly investigated.

## Results and discussion

**Figure 1:**
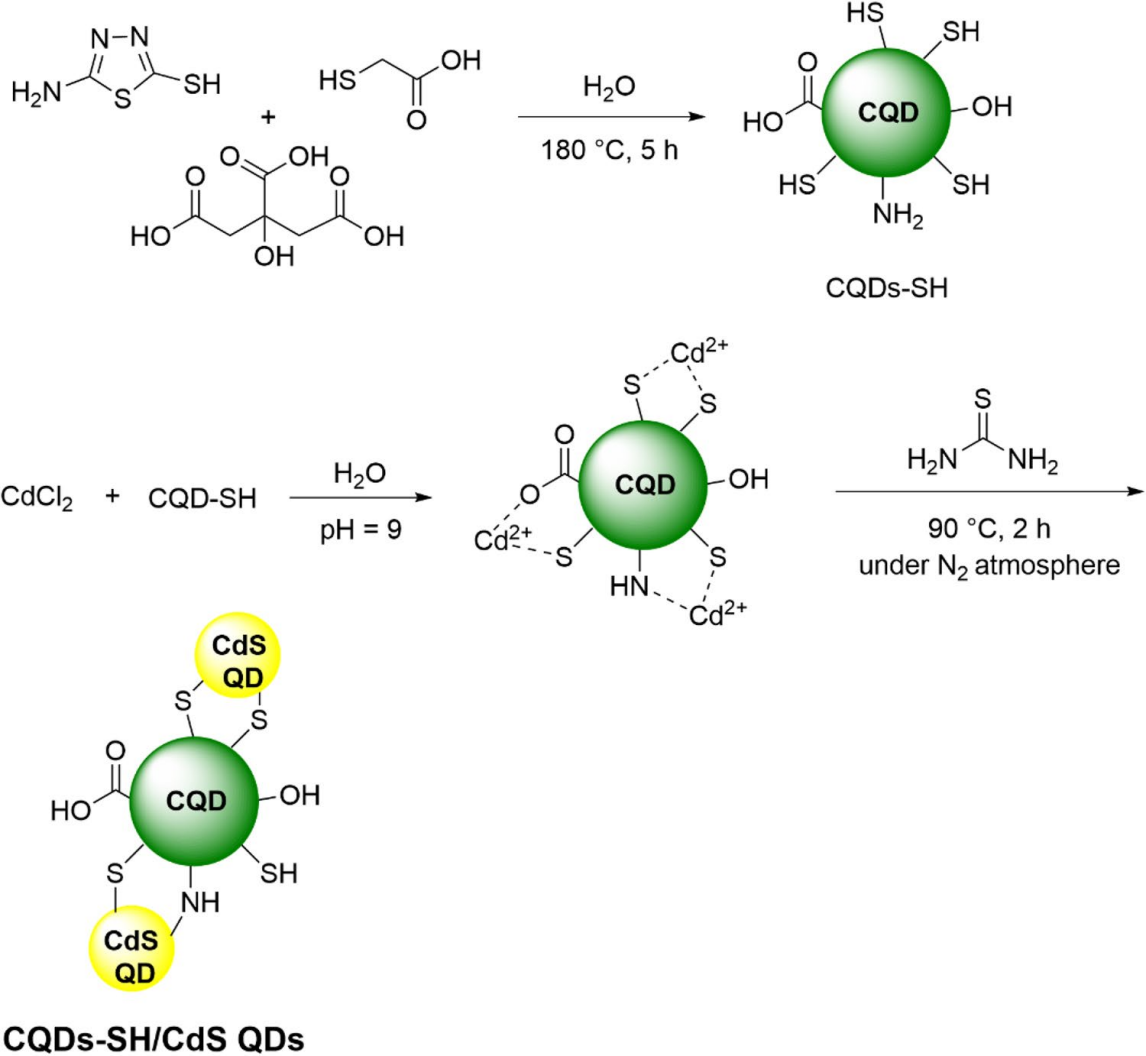
Schematic representation of synthesis procedure of CQDs-SH/CdS QDs.

Inspiring from recent developments in the stabilizing of QDs, we used CQDs containing thiol groups as a passivating agent for stabilization of CdS QDs. For this purpose, CQDs-SH were first prepared using a simple hydrothermal approach. Because of the existence of the potential coordination sites including –SH, –OH, –NH_2_ and –CO_2_H functional groups, the prepared CQDs-SH shows high coordination interaction towards Cd^2+^ ions. When the CQDs-SH and Cd^2+^, in basic medium, come in contact with thiourea (as source for sulfide ion), the S^2−^ ion binds with Cd^2+^, CdS QDs are synthesized and stabilized using CQDs-SH in aqueous solution. The presence of hydrophilic functional group (including –SH, –OH, –NH_2_ and –CO_2_H) on the surface of CQDs-SH imparts high dispersibility in water to CQDs-SH/CdS QDs. The stages of CQDs-SH/CdS QDs preparation are summarized in Fig. 1^18^.

### Characterizations

**Figure 2:**
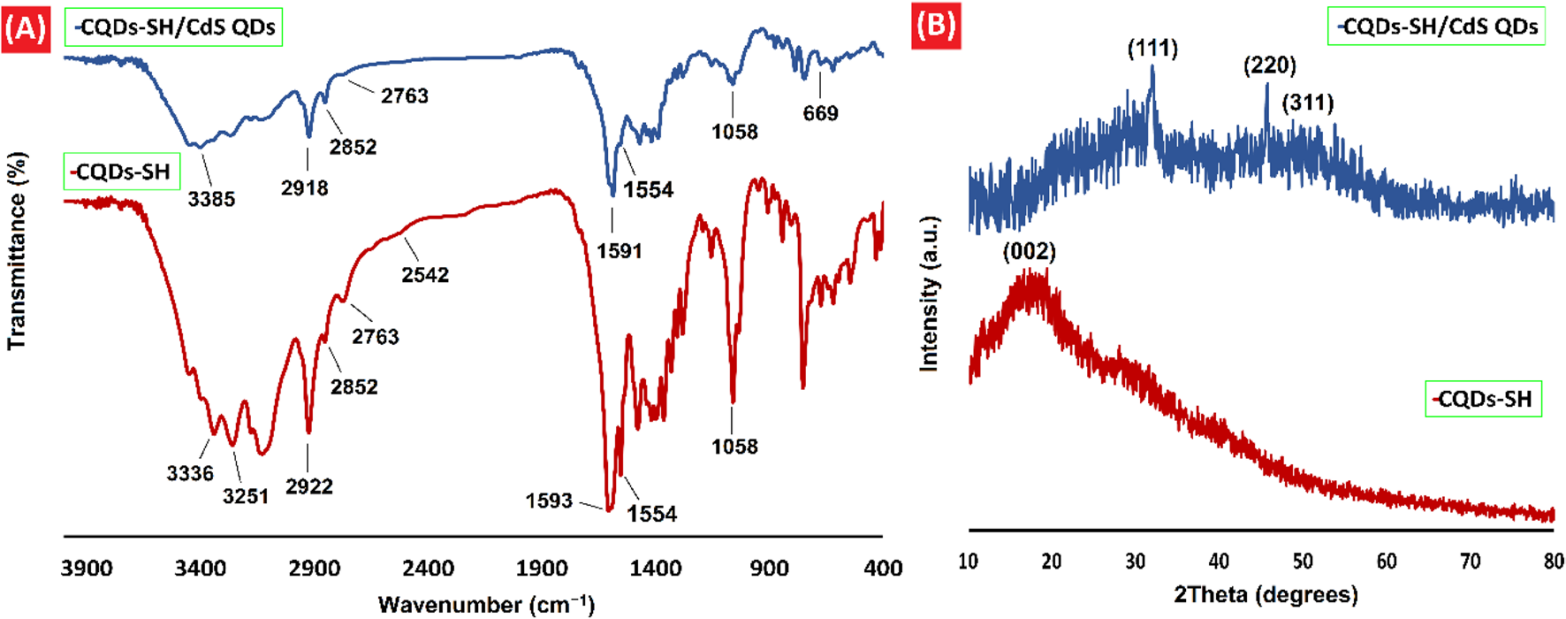
(**A**) FT-IR spectra of CQDs-SH and CQDs-SH/CdS QDs, (**B**) XRD patterns of CQDs-SH and CQDs-SH/CdS QDs.

Several distinct characterization techniques including FTIR, XRD, FESEM, EDX, TEM, HRTEM, XPS, Zeta, and fluorescence spectroscopy were employed to completely characterize as-synthesized CQDs-SH and CQDs-SH/CdS QDs. The FTIR spectrum obtained from CQDs-SH and CQDs-SH/CdS QDs are presented in Fig. 2A. In the case of CQDs-SH, the peaks at 3336 cm^-1^ and 3251 cm^-1^ are related to the O–H and N–H stretching vibrations. The peaks observed at 2922 cm^-1^ and 2852 cm^-1^ is attributed to the stretching vibrations—asymmetrical and symmetrical associated to the aliphatic C–H bonds. The peak at 2763 cm^-1^ and 2542 cm^-1^ were indicated the stretching vibration of the aromatic (from AMT compound) and aliphatic (from 2-MPA compound) S–H bonds on CQDs-SH, respectively^28,49^. The peak of 1593 cm^-1^ was indicated C=N stretching vibration, and the peak locating to 1554 cm^-1^ represented the N–H bending vibrations. The peak at 1058 cm^-1^ was designated as the C–S stretching vibrations^39,40^. Similarly, in the FTIR spectrum of CQDs-SH/CdS QDs, following functional groups were identified: OH and N–H stretching vibrations (a broad peak at 3385 cm^−1^), C–H bonds vibration (2918 and 2852 cm^−1^), C = N stretching vibration (1591 cm^−1^), N–H bending vibrations (1554 cm^−1^), C–S stretching vibrations (1058 cm^-1^). A diminished in the intensity of peak corresponding to the aromatic S–H bond (2763 cm^-1^) and disappearance of the 2542 cm^-1^ peak from aliphatic S–H bond units in the FTIR spectrum of CQDs-SH/CdS QDs can indicate most of the thiol groups of the CQDs-SH were successfully attached to the CdS QDs surface^28^. Besides, a band at 669 cm^-1^ is related to the presence of CdS QDs and attributed to Cd–S stretching^50^.

XRD analysis was performed to examine the structural parameters of CQDs-SH and CQDs-SH/CdS QDs. Figure 2B displays the XRD patterns of CQDs-SH and CQDs-SH/CdS QDs samples. The XRD pattern of CQDs-SH exhibits a single broad (002) peak centered at ~ 20°^51^. In addition, an extremely broad hump centered at ~ 27° was perceived in the XRD pattern of CQDs-SH/CdS QDs, corresponding to the (002) diffraction of CQDs. The peak of CdS QDs (111) was observed at 2θ values of 31.94° in this XRD pattern. Furthermore, the XRD pattern of CQDs-SH/CdS QDs shows diffraction peaks near 2θ = 45° and 51° which corresponded with (220) and (311) planes, respectively of CdS QDs^33^.

**Figure 3:**
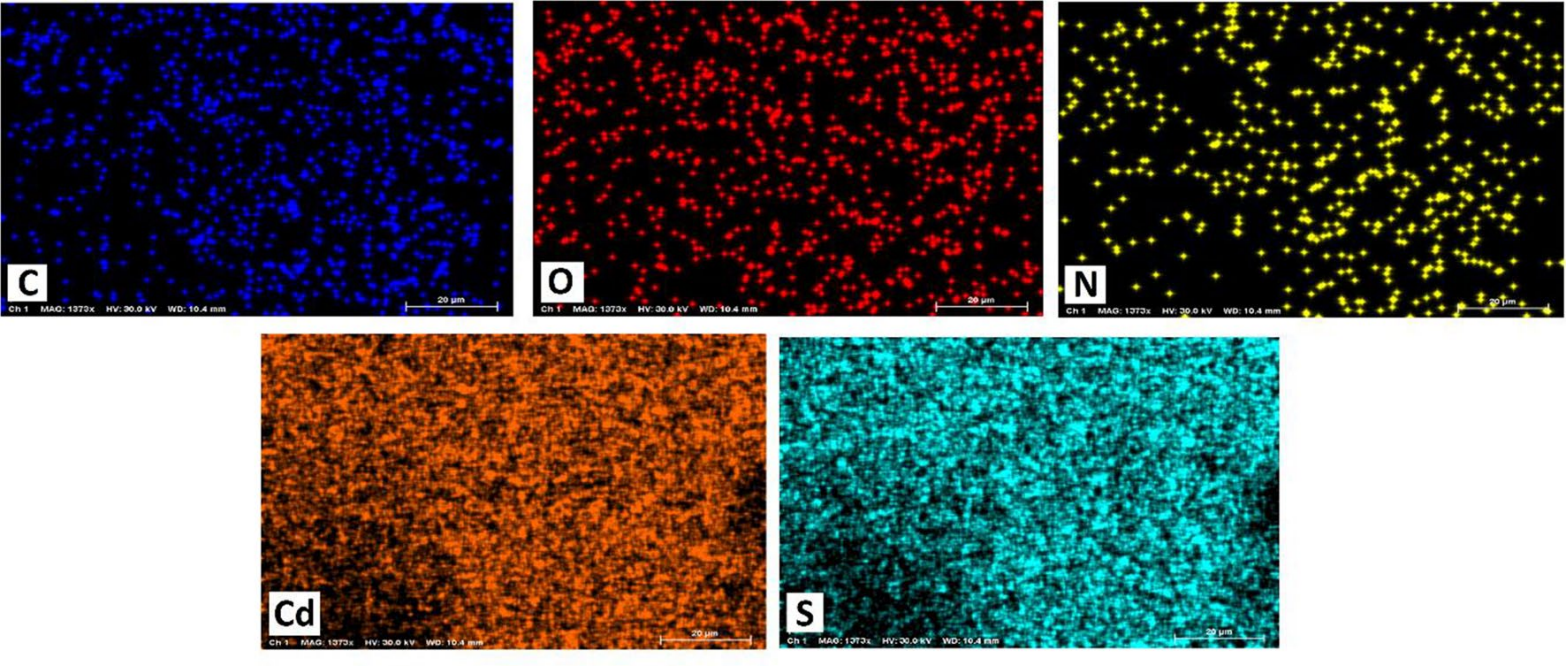
EDX mapping of CQDs-SH/CdS QDs.

EDX was employed to evaluate the elemental composition of CQDs-SH/CdS QDs. Supplementary Fig. 2 illustrates the EDX pattern of the synthesized sample. The EDX pattern indicates the presence of cadmium, sulfur, carbon, oxygen and nitrogen elements in CQDs-SH/CdS QDs. The elemental contents obtained from EDS are listed in Supplementary Table 1. The EDX elemental map images showing the homogeneity of all element’s distributions in the photocatalyst made out of CdS QDs and CQDs-SH (Fig. 3).

**Figure 4:**
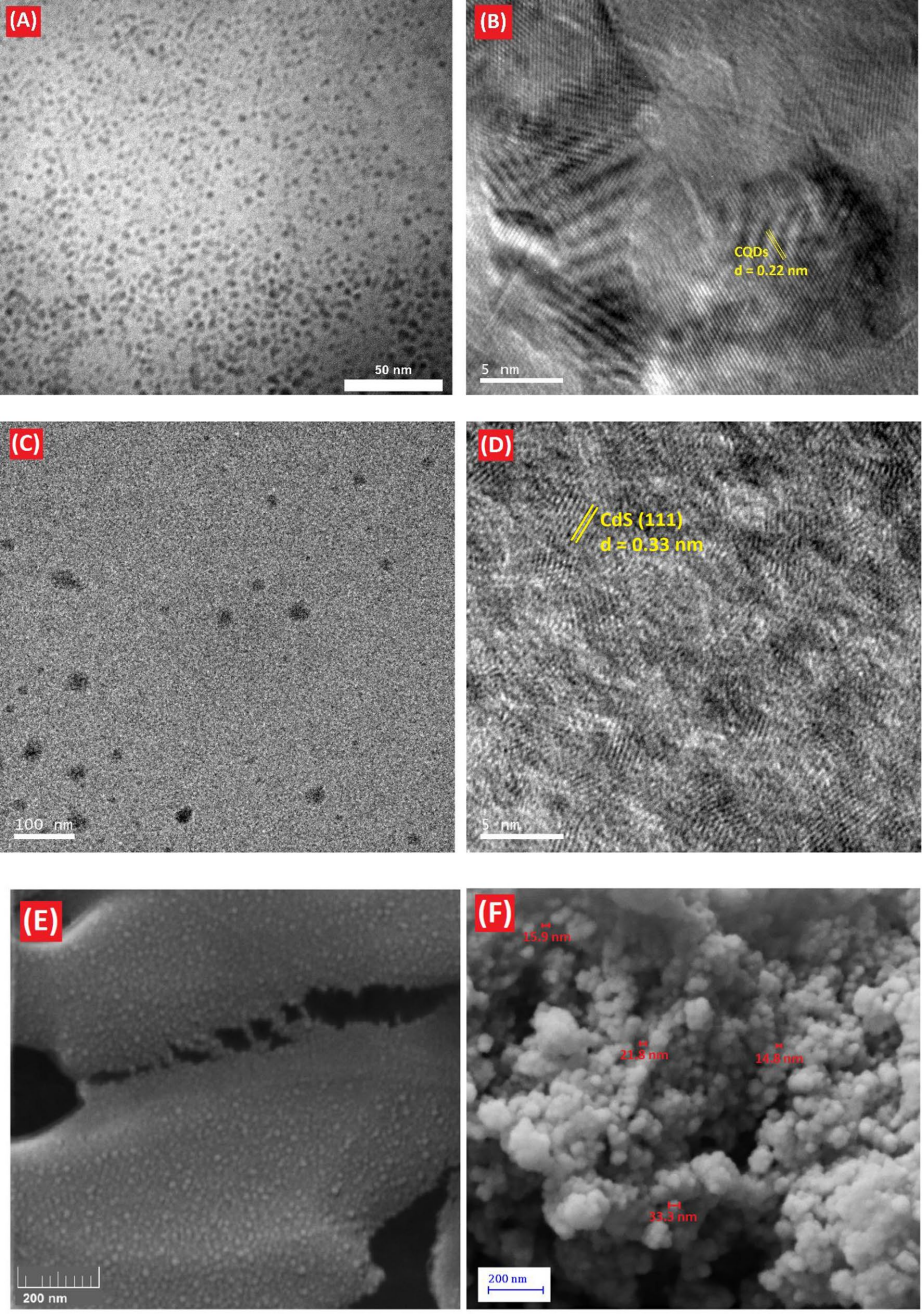
TEM and HRTEM images of (**A** and **B**) CQDs-SH, and (**C** and **D**) CQDs-SH/CdS QDs, respectively. FESEM images of (**E**) CQDs-SH, and (**F**) CQDs-SH/CdS QDs.

In this study, the size and morphology of CQDs-SH and CQDs-SH/CdS QDs nanocomposite particles was characterized using TEM. TEM images of the obtained CQDs-SH are shown in Fig. 4A. It can be observed that CQDs-SH predominantly possess an average size of 5 nm. Additionally, the crystalline structure of CQDs-SH is evident from the graphite lattice d-spacing of 0.22 nm (Fig. 4B)^52^. The TEM image clearly shows the presence of a nanocomposite consisting of CQDs-SH/CdS QDs with an average particle size ranging from 5 to 35 nm (Fig. 4C). The HRTEM analysis indicates that the CQDs-SH/CdS QDs nanocomposite contains well-dispersed, uniform, and small-sized CdS QDs with average diameters below 5 nm (Fig. 4D). This suggests that the CQDs containing –SH groups have the ability to stabilize and control the particle size of CdS QDs.

Furthermore, Fig. 4E and F display FESEM images of CQDs-SH and CQDs-SH/CdS QDs, respectively. The analysis reveals that the synthesized CQDs-SH exhibit spherical structures with sizes of up to 10 nm (Fig. 4E). Similarly, FESEM analysis of the synthesized CQDs-SH/CdS QDs clearly indicates the presence of uniform and small-sized CQDs-SH/CdS QDs (Fig. 4F).

**Figure 5:**
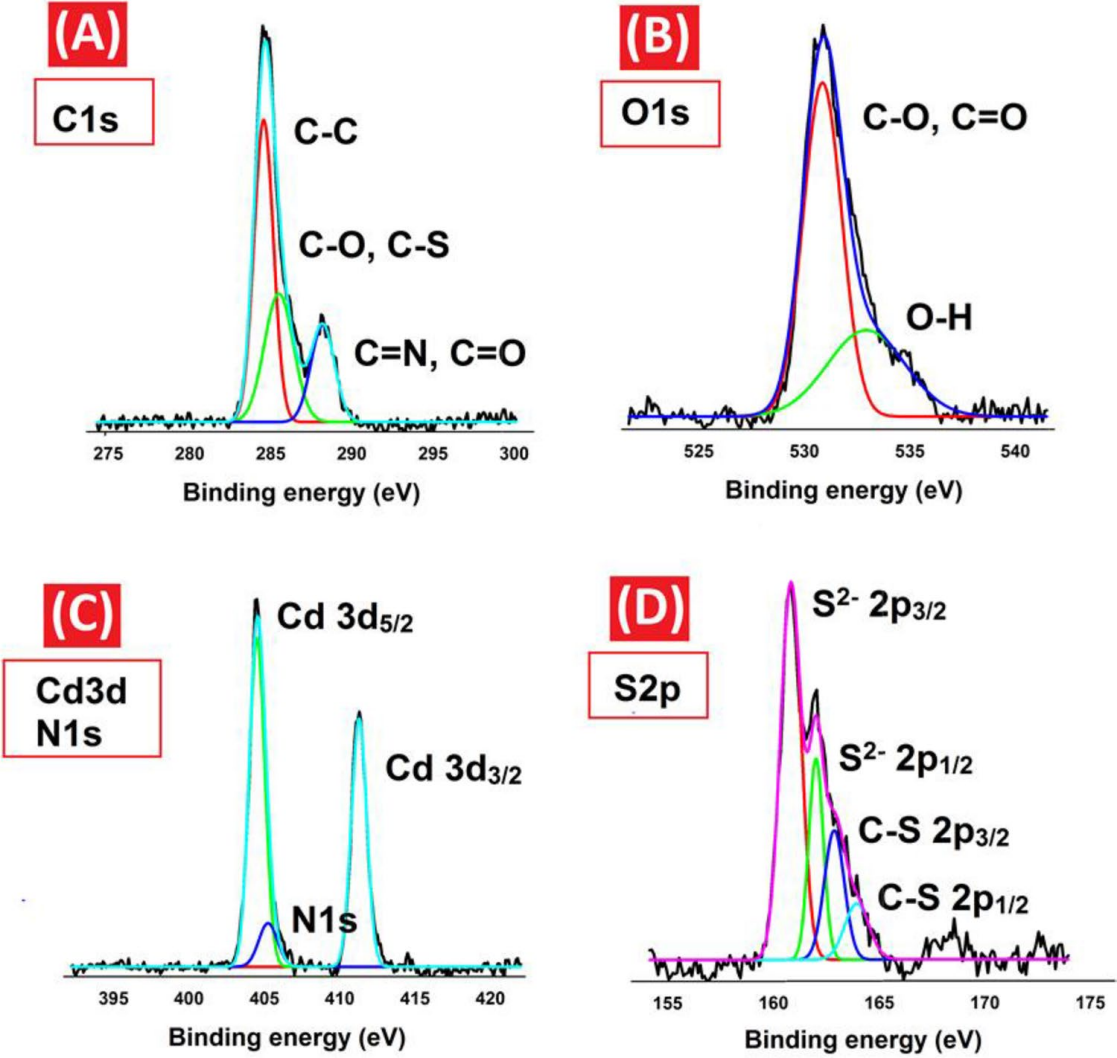
High resolution XPS spectra of (**A**) C 1 s, (**B**) O 1 s, (**C**) N 1 s and Cd 3d, and, (**D**) S 2p.

The chemical state of the CQDs-SH/CdS QDs nanocomposite was further characterized using XPS. The complete XPS spectra of CQDs-SH/CdS QDs clearly exhibit the presence of carbon (C), oxygen (O), sulfur (S), and cadmium (Cd) elements (Supplementary Fig. 3). In the C1s spectrum, peaks were observed at binding energies (BEs) of 284.5 eV (referring to C–C), 285.9 eV (referring to C–S and C–O), and 288.1 eV (referring to C=N and C=O) (Fig. 5A). The analysis of the O1s spectrum revealed peaks at BEs of 531.4 eV (assigned to C–O and C=O) and 533.6 eV (assigned to O–H) (Fig. 5B). Interestingly, in the case of nitrogen (N), the strong Cd 3d5/2 peak overlaps with the peak of N1s^53–55^. Fortunately, this issue of overlapping peaks can be resolved through proper peak fitting. Upon peak fitting, a distinct peak at 405.5 eV was observed, indicating the presence of nitrogen in the CQDs-SH/CdS QDs nanocomposite (Fig. 5C). Furthermore, the XPS spectrum for Cd demonstrates two sharp peaks at 404.9 and 411.9 eV, corresponding to Cd 3d5/2 and Cd 3d3/2, respectively (Fig. 5C). Moreover, the nanocomposite contains two types of bonds, Cd–S and C–S, which can be distinguished in the S 2p spectrum. The resolution spectra of S 2p were deconvoluted into four peaks. The Cd–S bonds exhibited peaks at 161.2 and 162.1 eV, corresponding to S 2p3/2 and S 2p1/2, respectively. Additionally, the C–S bonds were confirmed by two distinct peaks at 163.1 and 164.1 eV, corresponding to S 2p3/2 and S 2p1/2, respectively (Fig. 5D)^56^.

**Figure 6:**
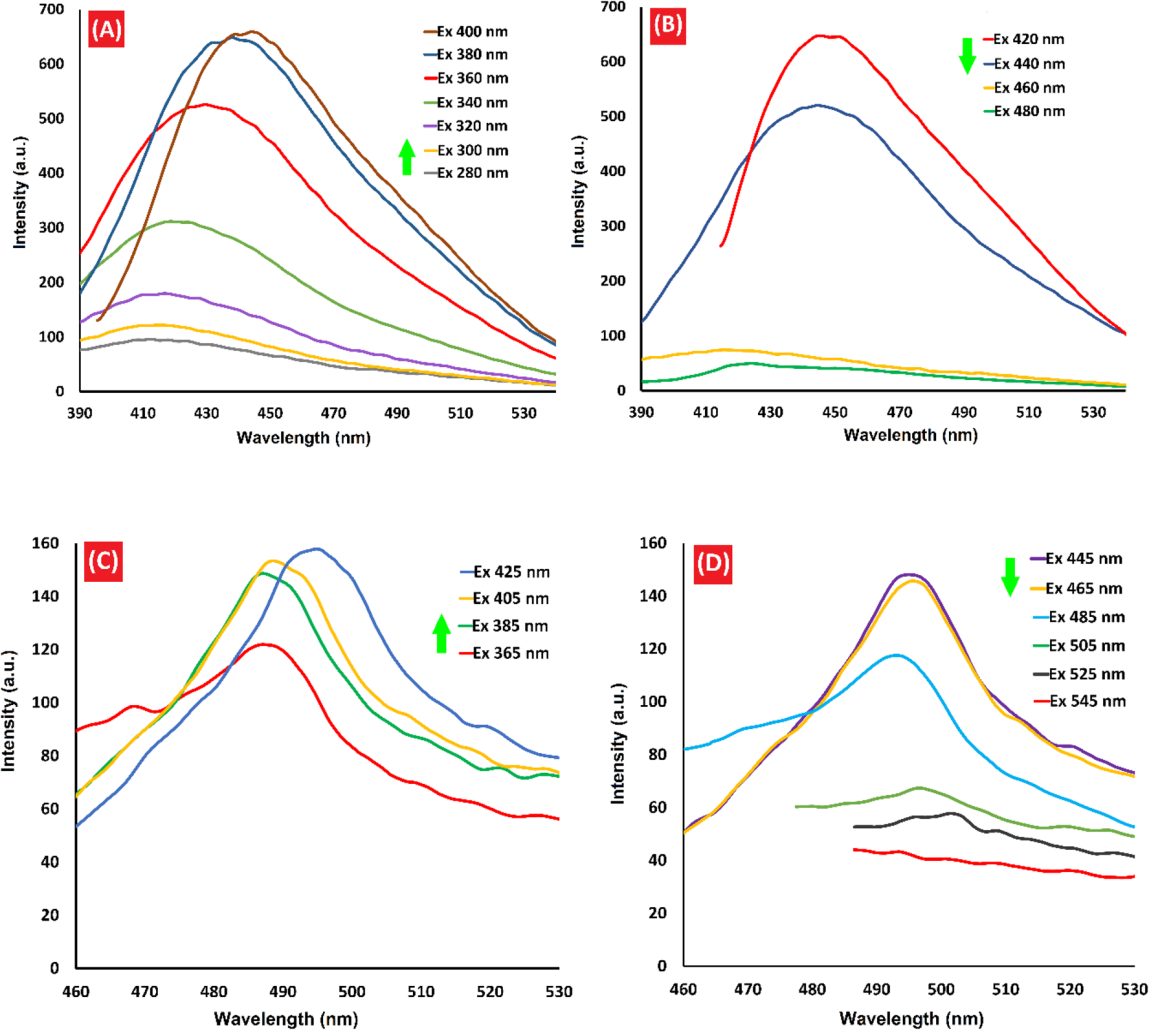
Fluorescence spectrum of (**A** and **B**) CQDs-SH, and (**C** and **D**) CQDs-SH/CdS QDs.

The emission wavelength and fluorescence intensity of QDs are dependent on their size. Thus, fluorescence spectroscopy serves as an effective tool for validating the optical properties of QDs. In the case of CQDs-SH, the fluorescence response was recorded at various excitation wavelengths ranging from 280 to 480 nm. As the excitation wavelength increased within this range, the fluorescence intensity of the CQDs-SH sample initially increased (Fig. 6A) and then decreased (Fig. 6B), consistent with previous research findings^40,42^. Notably, CQDs-SH exhibited strong emission centered at 445 nm when excited at 400 nm. Interestingly, the fluorescence profile of CQDs-SH/CdS QDs followed a similar pattern. Increasing the excitation from 345 to 525 nm resulted in an increase in the emission intensity, followed by a decrease (Fig. 6C and D). The strongest emission peak of CQDs-SH/CdS QDs, indicated by the blue line in Fig. 6C, was observed at 496 nm when excited at 425 nm. This red shift in the fluorescence emission can be attributed to the larger particle size of CQDs-SH/CdS QDs compared to CQDs-SH. Furthermore, supplementary Fig. 4 displays photographs of an aqueous dispersion of both CQDs-SH and CQDs-SH/CdS QDs under 365 nm UV irradiation. Under this UV irradiation, both samples exhibit blue emission^40^.

Zeta potential measurement was carried out to understand the stability and surface charge over CQDs-SH/CdS QDs. The measured zeta potential value of CQDs-SH/CdS QDs was estimated to be − 21.1 mV. The generally the higher zeta potential of quantum dots led to high stability in water. The zeta potential measurement of CQDs-SH/CdS QDs showed that there is a large negative charge at the surface, indicating that it is easier to attract holes and show high dispersion in water^57^.

### Photocatalytic degradation studies

**Figure 7:**
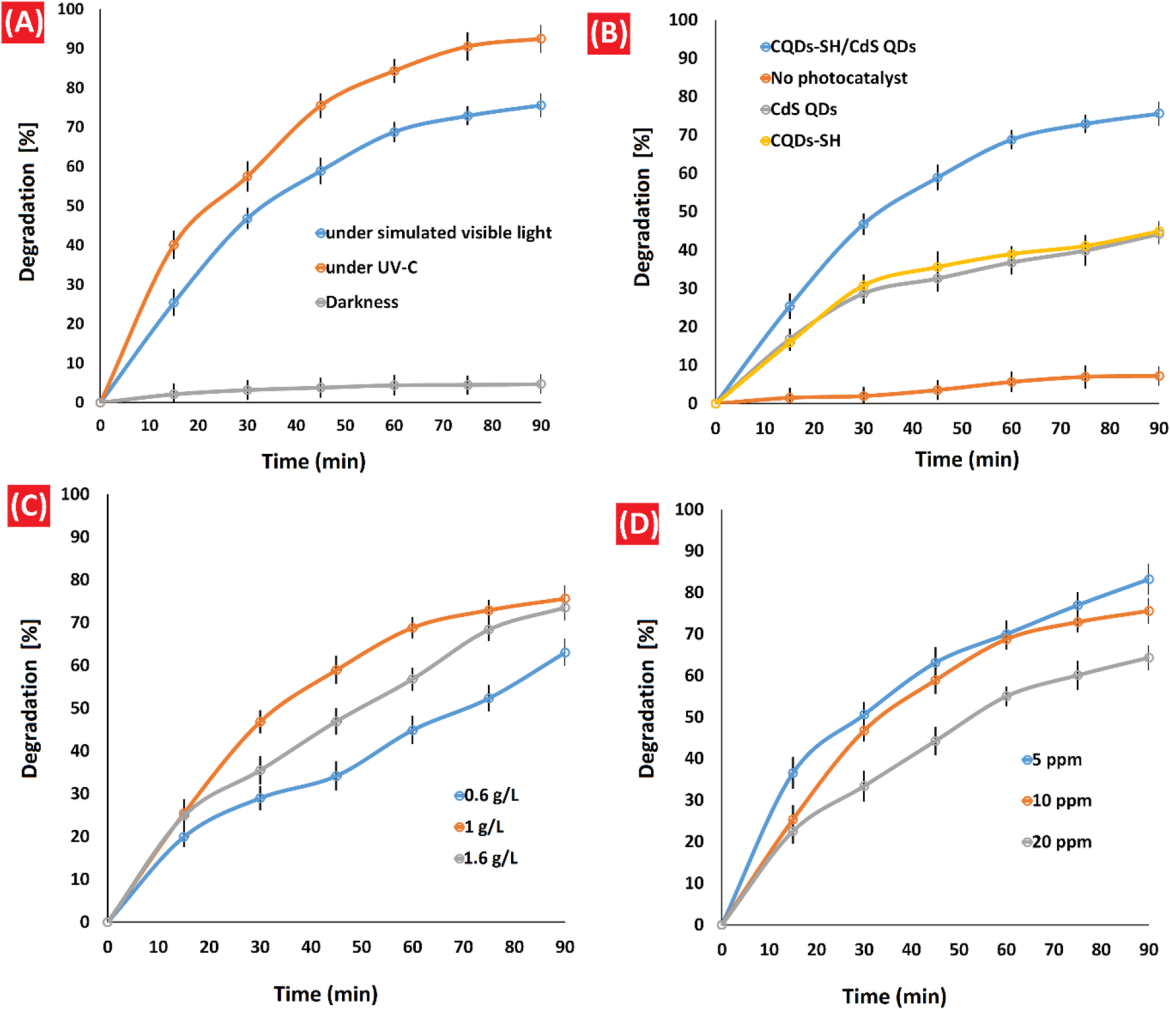
(**A**) Comparison of imidacloprid degradation under UV-C, simulated sunlight irradiation and darkness; Reaction conditions: pH = 7, [imidacloprid] = 10 ppm, at 25 °C and in the presence of 1 g/L of CQDs-SH/CdS QDs. (**B**) Comparison of imidacloprid degradation on different photocatalysts; Reaction conditions: under simulated visible light, pH = 7, [imidacloprid] = 10 ppm, at 25 °C and in the presence of 1 g/L of photocatalyst. (**C**) Effect of photocatalyst dosage on a degradation of imidacloprid; Reaction conditions: under simulated visible light, pH = 7, [imidacloprid] = 10 ppm, at 25 °C. and (**D**) Effect of initial imidacloprid concentration on the degradation; Reaction conditions: under simulated visible light, pH = 7, at 25 °C and in the presence of 1 g/L of CQDs-SH/CdS QDs.

To investigate the photocatalytic properties of the prepared sample (CQDs-SH/CdS QDs), we conducted a photodegradation reaction of imidacloprid in an aqueous solution. The results of the photocatalytic evaluation are summarized in Table 1. Efficient utilization of the full range of the solar light spectrum is a significant goal in the field of pollutant photodegradation. To achieve this objective, we examined the effect of different light sources, namely UV-C and simulated visible light, on the degradation of imidacloprid. As depicted in Fig. 7A, the photocatalytic performance of CQDs-SH/CdS QDs under UV-C irradiation was significantly higher compared to its performance under simulated visible light (92.20% degradation under UV-C irradiation vs 75.60% degradation under simulated visible light). Additionally, we performed a comparative experiment to evaluate the degradation of imidacloprid in the absence of light (dark conditions). Only a minor decrease in imidacloprid concentration (4.70%) was observed, which can be attributed to the adsorption of the pesticide on the photocatalyst sites. Since CQDs-SH/CdS QDs exhibited reasonable degradation percentages under simulated visible light, subsequent experiments were conducted under simulated visible light conditions. Next, we investigated the photocatalytic performance of the synthesized CQDs-SH/CdS QDs, as well as CQDs-SH and CdS QDs individually. Initially, the photodegradation process was carried out without the presence of a photocatalyst under simulated visible light, resulting in only a small percentage (7.22%) of degradation after 90 min. Subsequently, the degradation of imidacloprid was conducted using 1 g/L of the photocatalyst. Experimental observations revealed that the presence of photocatalysts significantly enhanced the percentage degradation of imidacloprid under simulated visible light, with the order of effectiveness being CQDs-SH/CdS QDs > CQDs-SH = CdS QDs. The CQDs-SH/CdS QDs composite exhibited the highest catalytic activity, achieving a 75.60% degradation of imidacloprid within 90 min, while CQDs-SH and CdS QDs achieved approximately 45% degradation (Fig. 7B and Table 1). As a result, the CQDs-SH/CdS QDs composite was chosen as the preferred photocatalyst for the degradation of imidacloprid.

**Table 1:** Photodegradation of the imidacloprid in aqueous solution.

Entry	Photocatalyst dose* (g/L)	Light source	Time (min)	Initial imidacloprid conc. (ppm)	pH	H_2_O_2_ (ppm)	Temperature (°C)	Imidacloprid removal (%)
1	–	Visible light	90	10	7	–	25	7.22
2	1	Visible light	90	10	7	–	25	44.93
3	1	Visible light	90	10	7	–	25	44.21
4	1	Visible light	90	10	7	–	25	75.60
5	1	UV– C	90	10	7	–	25	92.20
6	1	–	90	10	7	–	25	4.70
7	1	Visible light	60	10	7	–	25	68.81
8	1	Visible light	120	10	7	–	25	76.10
9	0.6	Visible light	90	10	7	–	25	63.01
10	1.6	Visible light	90	10	7	–	25	73.52
11	1	Visible light	90	5	7	–	25	83.22
12	1	Visible light	90	20	7	–	25	64.25
13	1	Visible light	90	10	5	–	25	58.45
14	1	Visible light	90	10	6	–	25	69.85
15	1	Visible light	90	10	8	–	25	81.23
16	1	Visible light	90	10	9	–	25	90.13
17	1	Visible light	90	10	7	10	25	81.85
18	1	Visible light	90	10	7	20	25	88.06
19	1	Visible light	90	10	7	40	25	91.87
20	1	Visible light	90	10	7	–	40	84.01
21	–	Visible light	90	10	9	–	25	11.06

*Photocatalyst = CQDs-SH (Entry 2), photocatalyst = CdS QDs (Entry 3), photocatalyst = CQDs-SH/CdS QDs (Entries 4–20).

According to the literature, CQDs exhibit moderate photocatalytic performance. The light absorption property of CQDs led to exciting electrons (e) and the transit from the valence band (vc) to the conduction band (cb) and this causes the generation of a positive hole (h_vb_^+^)/electron (e_cb_^−^) pairs. The photogenerated e-/h + pairs can react with water or hydroxyl groups to produce different reactive oxygen species such as ^∙^OH that play vital roles in the photocatalytic degradation processes. The following mechanism has been proposed for the photodegradation of organic compounds by CQDs under light irradiation^15^:

$$\begin{gathered} CQDs + h\nu \to e^{ - } + h^{ + } \hfill \\ {\text{H}}_{2} {\text{O }}or{\text{ OH}}^{ - } + h^{ + } \to ^{ \cdot } {\text{OH}} + {\text{H}}^{ + } \hfill \\ e^{ - } + {\text{O}}_{2} \to {\text{O}}_{2}^{{ \cdot - }} \hfill \\ Org \prec {\text{ H}} + h^{ + } \to Org \prec {\text{H}}^{{ + \cdot }} \leftrightarrow Or\dot{g} + {\text{H}}^{ + } \hfill \\ Org \prec {\text{H}} + ^{ \cdot } {\text{OH}} \to Or\dot{g} + {\text{H}}_{2} {\text{O}} \hfill \\ Or\dot{g} + {\text{O}}_{2} \to Org \prec {\text{ OO}}^{ \cdot } \xrightarrow{{{{{\text{O}}_{2} } \mathord{\left/ {\vphantom {{{\text{O}}_{2} } {{\text{H}}_{2} {\text{O}}}}} \right. \kern-\nulldelimiterspace} {{\text{H}}_{2} {\text{O}}}}}}Degradation \hfill \\ Org \prec {\text{H}} + ^{ \cdot } {\text{OH}} \to {\text{H}} \succ {\text{ }}Org \prec ^{ \cdot } {\text{OH}}\xrightarrow{{{{{\text{O}}_{2} } \mathord{\left/ {\vphantom {{{\text{O}}_{2} } {{\text{H}}_{2} {\text{O}}}}} \right. \kern-\nulldelimiterspace} {{\text{H}}_{2} {\text{O}}}}}}Degradation \hfill \\ \end{gathered}$$

CdS QDs also exhibit photocatalytic activity by generating hydroxyl radicals under light irradiation. Previous studies have shown that CdS QDs/CQDs composites have higher light absorption capacity and more efficient electron transfer compared to CdS QDs alone^18^. Furthermore, the influence of reaction time on the percentage of imidacloprid photodegradation within the range of 15–120 min was investigated. The highest degradation rate of imidacloprid was observed during the initial 30 min, followed by a slower degradation rate in the subsequent 60 min. Increasing the irradiation time from 90 to 120 min did not result in a significant change in the percentage of pesticide degradation. Therefore, a reaction time of 90 min was chosen for the photodegradation experiments (Table 1). At the beginning of the degradation process, the high concentration of imidacloprid leads to a rapid binding to photocatalyst sites, which may contribute to the initially observed higher degradation rates. However, as the reaction progresses, the concentration of intermediate products increases, potentially leading to competition between imidacloprid and these intermediates for degradation. These factors could contribute to a decrease in the rate of photocatalytic degradation of imidacloprid^17,22^.

### Effect of photocatalyst concentration

The amount of CQDs-SH/CdS QDs photocatalyst has a significant influence on the percentage of pesticide photodegradation. Previous studies have reported mixed effects of the photocatalyst amount on the photodegradation percentage. In our investigation, we examined the impact of varying concentrations of CQDs-SH/CdS QDs (ranging from 0.6 to 1.6 g/L) on the photodegradation percentage of imidacloprid (Fig. 7C). The results demonstrated that the degradation rate of imidacloprid increased as the amount of CQDs-SH/CdS QDs increased from 0.6 to 1 g/L. However, a slight decrease in the percentage degradation was observed when the amount of CQDs-SH/CdS QDs was further increased to 1.6 g/L. These findings align with expectations. Initially, as the catalyst amount increased, the rate of photodegradation accelerated due to the availability of more photocatalyst sites. However, once the catalyst amount exceeded a certain threshold, the solution became more opaque, impeding the penetration of photon flux. Consequently, the photodegradation rate decreased. Therefore, the quantity of CQDs-SH/CdS QDs significantly affects the percentage of pesticide photodegradation. While an increase in catalyst amount initially enhances the degradation rate, excessive quantities can lead to reduced degradation due to reduced photon flux penetration caused by increased solution opacity.

### Imidacloprid concentration effect

The impact of different initial pesticide concentrations on the percentage of photodegradation was studied by changing the concentration from 5–20 ppm (Fig. 7D). It is evident in Fig. 7D, that the degradation rate is reduced with an increase in the concentration of the pesticides. Such an effect is expected because, with an increase in the pesticide concentrations, the concentration ratio of available active photocatalysts sites to pollutant molecules decreases which in turn leads to a decrease in the degradation of the pesticide.

### Effect of pH

**Figure 8:**
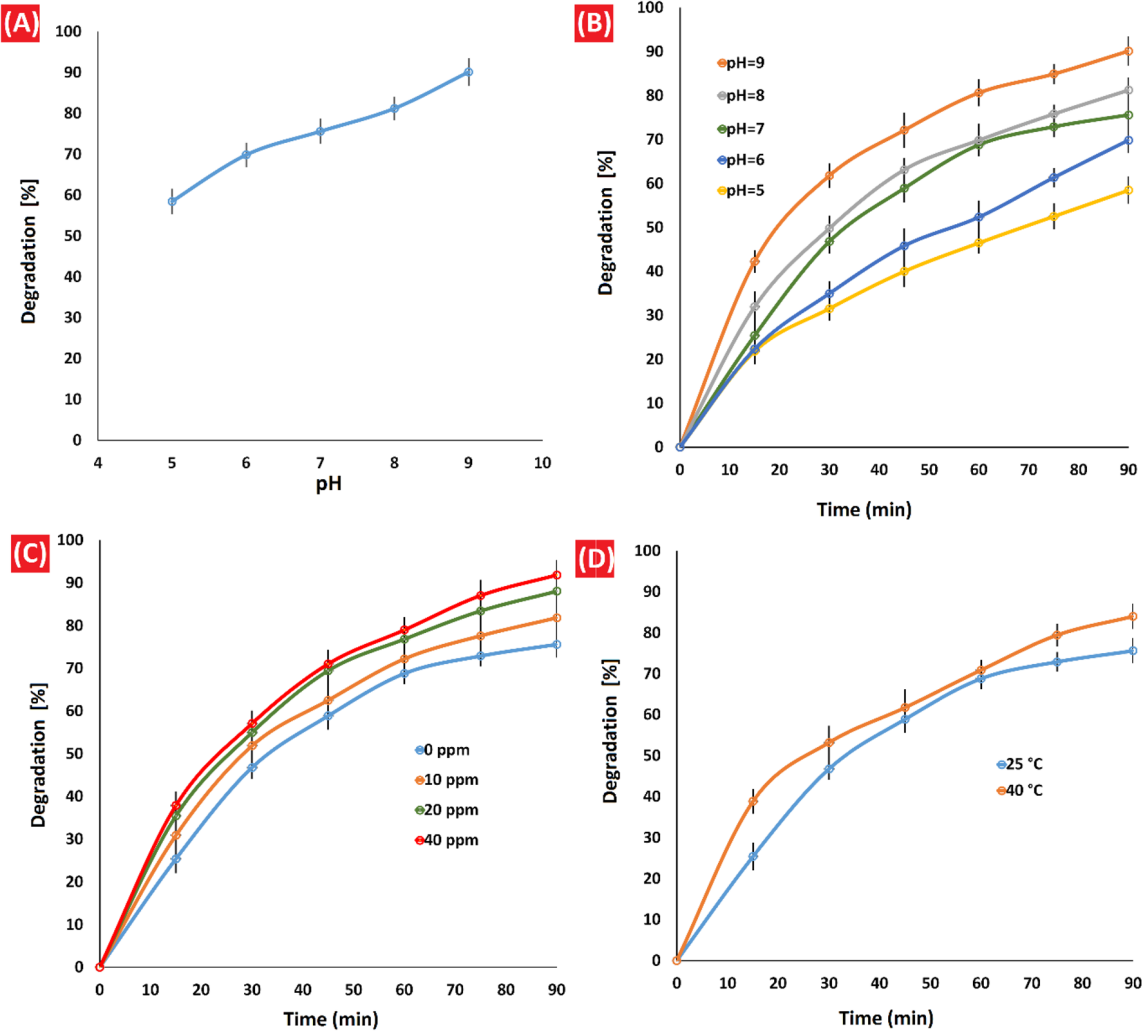
(**A** and **B**) Effect of pH on imidacloprid degradation; Reaction conditions: under simulated visible light, [imidacloprid] = 10 ppm, at 25 °C and in the presence of 1 g/L of CQDs-SH/CdS QDs. (**C**) Comparison of imidacloprid degradation in the presence and absence of H_2_O_2_ (0–40 ppm); Reaction conditions: under simulated visible light, pH = 7, [imidacloprid] = 10 ppm, at 25 °C and in the presence of 1 g/L of CQDs-SH/CdS QDs, (**D**) Effect of reaction temperature on imidacloprid degradation; Reaction conditions: under simulated visible light, pH = 7, [imidacloprid] = 10 ppm and in the presence of 1 g/L of CQDs-SH/CdS QDs.

Based on literature review, the impact of pH on the degradation rate is not well understood, and conflicting results have been reported. Some studies have demonstrated higher removal percentages of imidacloprid under alkaline conditions for certain photocatalysts, such as ZnO/CoFe_2_O_4_ and GO/Fe_3_O_4_/TiO_2_-NiO photocatalyses^58,59^. Conversely, other studies have shown higher imidacloprid photodegradation percentages under acidic conditions^60,61^. Considering the crucial role of pH in the photodegradation process, we investigated the influence of initial pH on the efficiency of imidacloprid photodegradation at pH values of 5, 6, 7, 8, and 9 (Table 1 and Figs. 8A and B). Our findings revealed a strong correlation between the pH of the solution and the rate of imidacloprid photocatalytic degradation. We observed a remarkable increase in the rate of imidacloprid degradation (up to 90.13%) at pH 9. Conversely, in acidic media (pH 5 and 6), the degradation of imidacloprid was higher at neutral pH than at acidic pH, and a sudden drop in degradation was observed (58.45% degradation at pH 5).

To clarify this result, a graph between pH and zeta potential (Supplementary Fig. 5) was used to determine the pH value in the zero point of charge (pH_zpc_). The dissociation constant (pKa) value of imidacloprid is 11.12 and the pH_zpc_ values for CQDs-SH/CdS QDs was equal to 5.2^62^. Therefore, the photocatalyst surface has a negative charge at the pH values greater than pH_zpc_^63,64^ and on the other hand, imidacloprid has a positive charge at pH values less than 11.12. As a result it is expected that imidacloprid well absorbed on surface of the photocatalyst at pH above 5.2, in turn, will take part in the reaction with the radicals in the solution, therefore, imidacloprid degradation is highest under alkaline conditions^62^.

To gain a better understanding of the influence of alkaline conditions on the photodegradation process, we investigated the extent of imidacloprid degradation at pH 9 under simulated visible light in the absence of photocatalyst (Table 1). The results showed only 11.06% photodegradation of imidacloprid, which can be attributed to alkaline hydrolysis. Under neutral and acidic conditions, imidacloprid exhibits high stability, but it undergoes hydrolysis in alkaline conditions, leading to the formation of the main reaction product, 1-[(6-chloro-3-pridinyl)methyl]-2-imidazolidone. Therefore, the influence of pH on the photodegradation process is complex and depending on the specific conditions and photocatalysts involved^65^.

### Effect of oxidizing agent

It has been shown that hydrogen peroxide, in low concentrations, enhances the photodegradation process efficiency through producing hydroxyl radicals in combination with the UV irradiations and inhibition of electron–hole pair recombination. However, increasing the concentration of H_2_O_2_ has a detrimental effect on degradation due to hydroxyl radicals-scavenging activity of H_2_O_2_ in high concentrations^66,67^.

The investigation on the photodegradation of imidacloprid by CQDs-SH/CdS QDs in the presence of H_2_O_2_ (0–40 ppm) showed that as the loading of H_2_O_2_ was increased from 0 to 20 ppm the extent of photodegradation percentage of imidacloprid increased from 75.60 to 88.06% while it increased to 91.87% for the 40 ppm loading of H_2_O_2_. It can be seen that smaller increases were observed as the loading of H_2_O_2_ was increased from 20 to 40 ppm, it can be explained considering the fact that at high concentrations H_2_O_2_ may act also as a hydroxyl radical scavenger (Fig. 8C).

### Effect of reaction temperature

The effect of temperature on the reaction was also studied. It was observed that the photodegradation of imidacloprid is increased with increasing temperature from 25 to 40 °C (Fig. 8D). When the temperature increases from 25 to 40 °C, percentage degradation of imidacloprid increases up to 84%. Our findings are in agreement with previous studies^59^. As the temperature increases, the charge carrier mobility and interfacial charge transfer improve so that the photocatalytic activity increases^68^.

**Figure 9:**
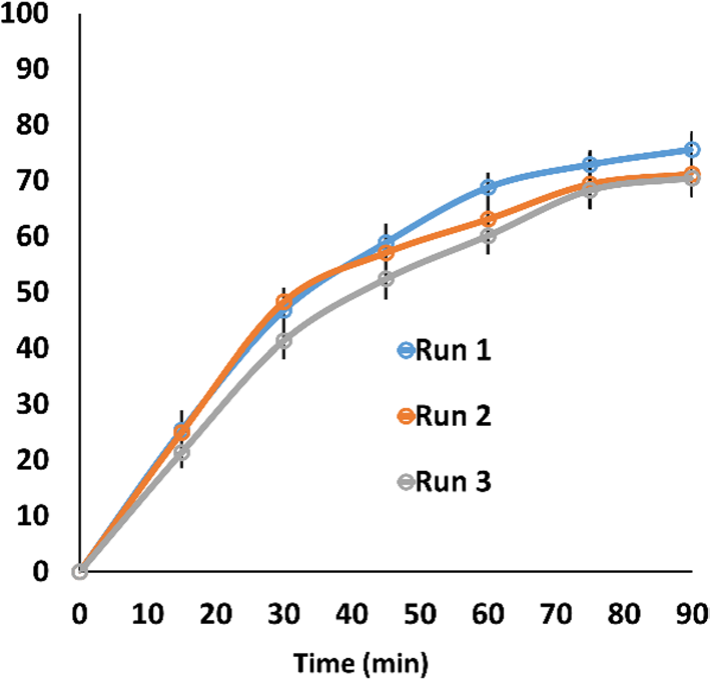
Photodegradation of imidacloprid with CQDs-SH/CdS QDs under three recycling runs.

Other important point to be addressed is reusability of the photocatalysts. Therefore, we have investigated stability and recycling potential of the CQDs-SH/CdS QDs. The results of repetitive photodegradation of imidacloprid in water under simulated visible light irradiation during three consecutive cycles are presented in Fig. 9. After each cycle, the photocatalyst was separated by using centrifugation, washed twice with distilled water and a fresh solution of imidacloprid pesticide with the same concentration was refilled in the next photocatalytic run. The CQDs-SH/CdS QDs photocatalyst was recycled three times and was found to exhibit slight decreases in photocatalytic efficiencies. According to these results, it can be concluded that CQDs-SH/CdS QDs photocatalyst display good stability in the photocatalytic process.

The quantitative analysis of imidacloprid and photodegradation products have been evaluated by HPLC (Supplementary Fig. 6). Supplementary Fig. 7 display the HPLC chromatogram of imidacloprid (10 ppm) as a standard solution. The retention time for imidacloprid was found to be around 5.08 min. The results of the HPLC analysis revealed that there were three intermediates existing in the photodegradation process in the presence of CQDs-SH/CdS QDs as photocatalyst. The photodegradation products appeared at retention time of around 1.82 min, 2.95 min and 3.72 min in the HPLC chromatogram (Supplementary Fig. 7).

**Figure 10:**
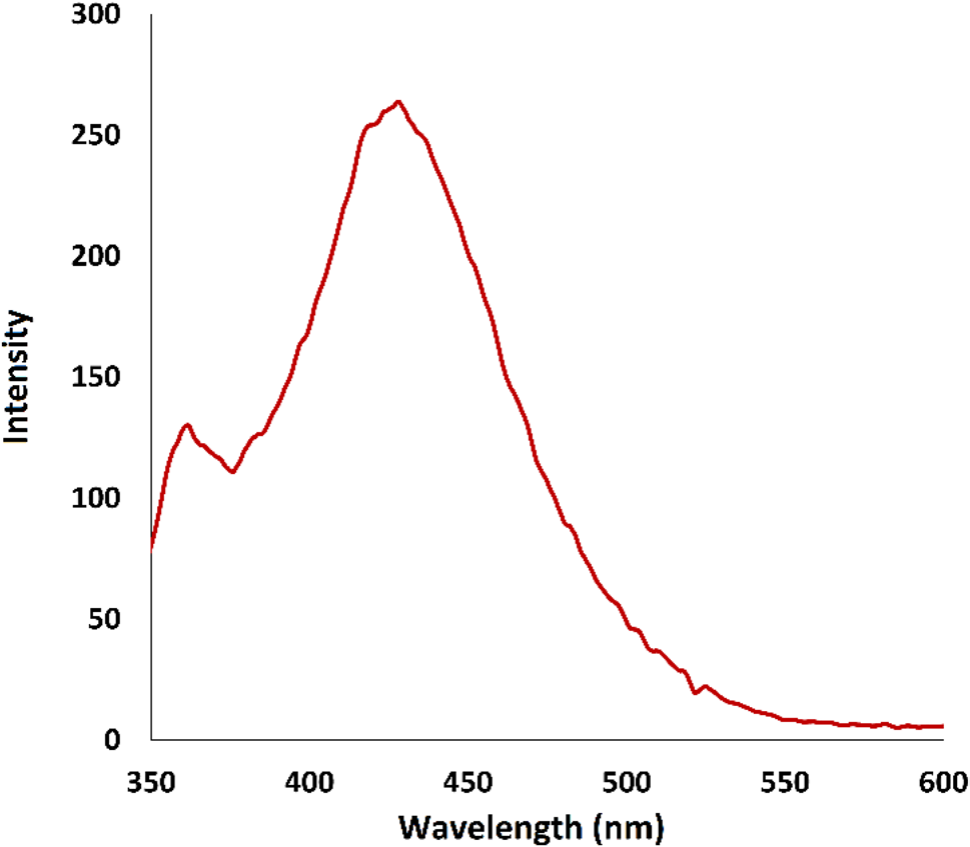
Fluorescence spectra of TA solution (excited at 315 nm), in the presence of CQDs-SH/CdS QDs at 90 min irradiation.

Studies have demonstrated that the active oxygen species such as •OH play vital roles in the photocatalytic degradation processes, therefore, the photocatalytic performance of the materials depended on their •OH production efficiency^69^. In this regard, significant photodegradation percentage of insecticide imidacloprid in aqueous solution was achieved with CQDs-SH/CdS QDs nanocomposite which this suggests that high production of hydroxyl radicals in the presence of CQDs-SH/CdS QDs. The measurement of OH radicals dissolved in water during the photocatalytic reaction in the presence of CQDs-SH/CdS QDs was studied using terephthalic acid (TA) as an OH radical scavenger. Studies have found that the OH radical can convert TA to hydroxy terephthalic acid (HTA) which emits light at λ = 425 nm while TA does not^70^, therefore the fluorescent intensity of TA solution is directly proportional to the amount of ˙OH production during the photocatalytic process. Therefore, we have monitored the fluorescent intensity of TA in the presence of CQDs-SH/CdS QDs at 90 min irradiation under simulated visible light (Fig. 10). The high fluorescence intensity at 90 min irradiation suggests that the high concentration of hydroxyl radicals have been generated during the photocatalytic reaction in the presence of CQDs-SH/CdS QDs^71^.

Production of hydroxyl radicals and thus effective significant photodegradation of insecticide imidacloprid in the presence of CQDs-SH/CdS QDs can be explained as follows:

The energy of the emitted light is responsible for electrons promotion from the valence band (vb) to the conduction band (cb), as a result of this positive hole (h_vb_^+^) and the electron (e_cb_^−^) are generated. Subsequently, h_vb_^+^ and e_cb_^−^ react with water or hydroxyl groups to produce highly reactive oxygen species such as hydroxyl radical^72,73^.

**Figure 11:**
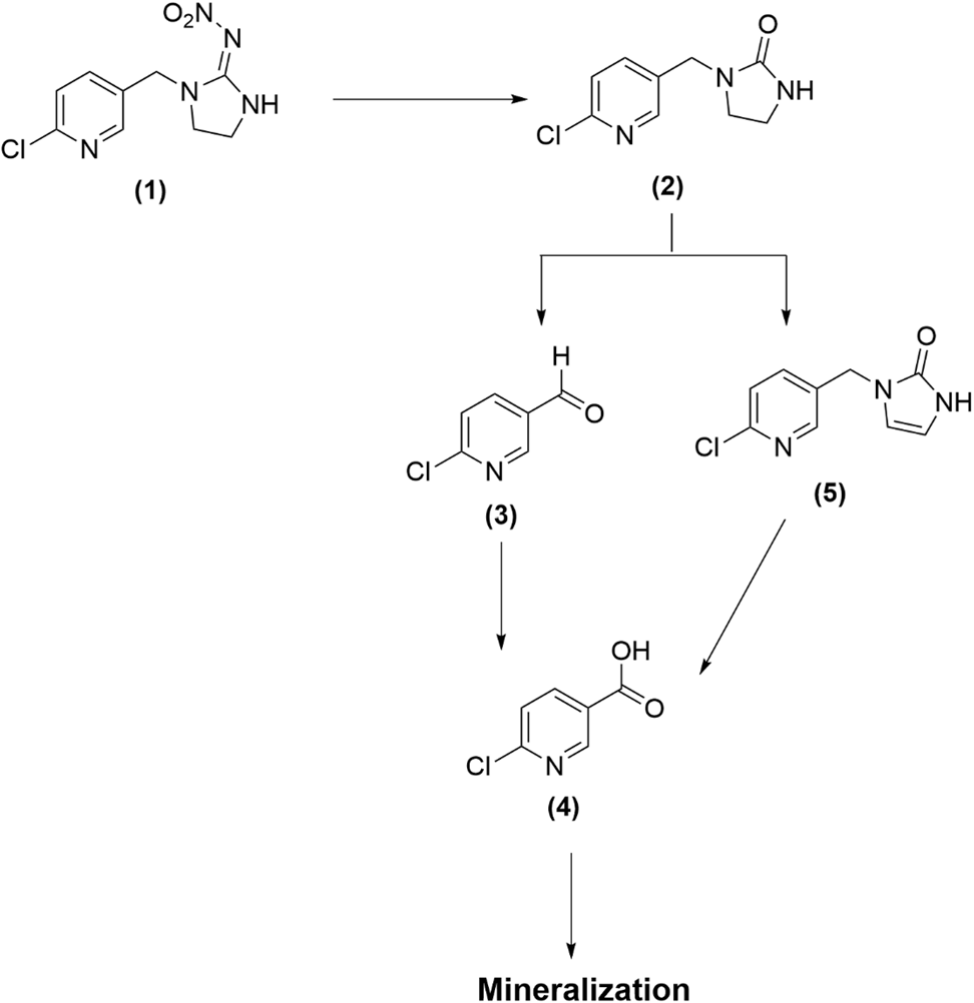
Proposed photodegradation pathway of imidacloprid.

The imidacloprid degradation products were also detected by liquid chromatography-mass spectrometry (LC–MS) (Supplementary Fig. 8). The compound responsible for the peaks at 2.45 min (m/z = 142) is designated as a photodegradation product of imidacloprid. According to the mass spectral data obtained from the chromatographic peak at 2.45 min (m/z = 142), 6-chloro nicotinaldehyde is considered as the main photoproduct of the degradation process (Supplementary Figs. 8A and 9). However, imidacloprid-olefin (m/z = 209) and 6-chloronicotinic acid (m/z = 159), and an unknown photoproduct (m/z = 180) were also detected at lower concentrations. It was therefore concluded that the photodegradation process of imidacloprid here mostly occurred in the imidazolidine fraction, and the structure of the chloropyridine ring would be less photodegraded^74^. In addition, the undegraded imidacloprid was also detected at 3.58 min (m/z = 257) (Supplementary Figs. 8B and 10). As a result, the photodegradation pathway for imidacloprid has been suggested based on LC–MS result and previous research (Fig. 11)^75^. The degradation of imidacloprid is happened through attack of photo-generated hydroxyl radicals on imidacloprid molecule and intermediate species are subsequently formed. The hydrolysis of imidacloprid (**1**) could result in the formation of imidacloprid-urea (**2**). It should be noted that photo hydrolysis of imidacloprid will be higher in alkaline conditions^76^. Since we observed an impressive increase in the rate of the photocatalytic degradation of imidacloprid in alkaline conditions in the presence of CQDs-SH/CdS QDs, the photodegradation process presumably proceeds through formation of imidacloprid-urea (**2**). The imidacloprid-urea compound (**2**) may degrade directly to 6-chloro nicotinaldehyde (**3**). The 6-chloronicotinic acid (**4**), the final product of photodegradation of imidacloprid reported in many studies, can be formed during oxidation of 6-chloro nicotinaldehyde (**3**)^76^. Subsequently, this intermediate can convert into smaller degradation products by hydroxyl radical attack.

Table 2 lists the comparison of maximum photodegradation of imidacloprid from aqueous solution using various photocatalysts. Compared with some data in the literature, Table 2 shows that the CQDs-SH/CdS QDs studied in this work has extremely high photocatalytic activity.

**Table 2:** Comparison of the efficiency of the synthesized photocatalyst with some previously reported photocatalysts for photodegradation of imidacloprid.

Entry	Photocatalyst	Reaction conditions	Imidacloprid removal (%)	References
1	ZnO/CoFe_2_O_4_ magnetic nanocomposite	[Imidacloprid] = 5 ppm, ZnO/CoFe_2_O_4_ = 10 g/L, pH = 10, r.t., 0.75 h	100	^58^
2	H_3_PW_12_O_40_/TiO_2_-In_2_S_3_	[Imidacloprid] = 8 ppm, H_3_PW_12_O_40_/TiO_2_-In_2_S_3_ = 2 g/L, visible light (λ = 400 nm), 5 h	82.7	^77^
3	GO/Fe_3_O_4_/TiO_2_-NiO	[Imidacloprid] = 10 ppm, GO/Fe_3_O_4_/TiO_2_-NiO = 26 g/L, pH = 9, visible light, 0.5 h	97.34	^59^
4	g-C_3_N_4_/ZnO (20:80)	[Imidacloprid] = 10 ppm, g-C_3_N_4_/ZnO = 0.6 g/L, UV-C light, 0.5 h	95.6	^78^
5	P-doped g-C_3_N_4_/fullerene (C60/PCN)	[Imidacloprid] = 25.5 ppm, [H_2_O_2_] = 1.5 × 10^–4^ mol/L, C60/PCN = 0.6 g/L, pH = 4, Light intensity = 750 lx., r.t., 6 h	95	^79^
6	Zn_0.1_Cd_0.9_S/SnIn_4_S_8_	[Imidacloprid] = 5 ppm, Zn_0.1_Cd_0.9_S/SnIn_4_S_8_ = 0.2 g/L, visible light, 4 h	55	^80^
7	Ag_2_O/g-C_3_N_4_	[Imidacloprid] = 10 ppm, Ag_2_O/g-C_3_N_4_ = 1 g/L, visible light, 2 h	80	^81^
8	This work	[Imidacloprid] = 10 ppm, CQDs-SH/CdS QDs = 1 g/L, visible light, pH = 9, 1.5 h	90.13	

## Conclusions

In conclusion, novel designed carbon quantum dots containing thiol groups (CQDs-SH) were successfully synthesized using a simple hydrothermal approach and were used as a passivating agent for stabilization of CdS QDs. It was expected that the well-designed composite structure leads to dramatically improved photocatalytic performance. The photocatalytic activity of the nanocomposite was assessed through photodegradation of insecticide imidacloprid from water. In the present work, the effect of numerous parameters for understanding their possible impacts on imidacloprid degradation has been investigated. We found strong dependence of pH of the solution on the degradation rate of imidacloprid degradation in the presence of CQDs-SH/CdS QDs. We perceived maximum degradation (up to 90.13%) under highly basic conditions (pH 9). We believe this work can open up a new way for designing and preparing efficient nanocomposites photocatalysts for organic pollutants degradation.

### Supplementary Information

Supplementary Information.

## Data Availability

All the associated with this work are presented here (and its Supplementary Information file) and further will be made available on reasonable request. Correspondence and requests for materials should be addressed to A.R.
