# Supplementary material for: Photocatalytic removal of imidacloprid pesticide from wastewater using CdS QDs passivated by CQDs containing thiol groups
Source: Sci Rep. 2024 Jan 4;14:530. doi: 10.1038/s41598-023-49972-8 (PMC10766997; doi:10.1038/s41598-023-49972-8)
Supplement: Supplementary file 1 — Supplementary Information. [file 41598_2023_49972_MOESM1_ESM.docx]

**Photocatalytic Removal of Imidacloprid Pesticide from Wastewater Using CdS QDs Passivated by CQDs Containing Thiol Groups**

Homa Targhan,^€^ Aram Rezaei,^€^* Alireza Aliabadi,^£^* Ali Ramazani,^¥^* Zhefei Zhao,^§Þ^ and Huajun Zheng ^§Þ^*

€ Nano Drug Delivery Research Center, Health Technology Institute, Kermanshah University of Medical Sciences, Kermanshah, Iran

£ Pharmaceutical Sciences Research Center, Health Institute, School of Pharmacy, Kermanshah University of Medical Sciences, Kermanshah, Iran

¥ Department of Chemistry, University of Zanjan, Zanjan 45371-38791, Iran

§ Petroleum and Chemical Industry, Key Laboratory of Organic Electrochemical Synthesis, Zhejiang University of Technology, Hangzhou 310032, China

Þ Department of Applied Chemistry, Zhejiang University of Technology, Hangzhou 310032, China


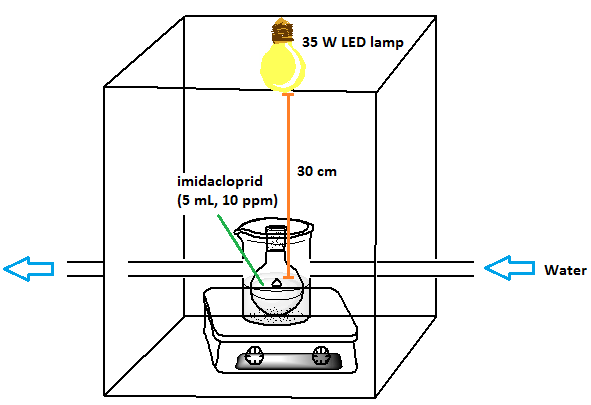


**Supplementary figure 1.** The scheme of the experimental set-up

**Supplementary table 1.** The element content table based on EDX data

| Element | Mass Norm. [%] | Atom [%] |
| --- | --- | --- |
| Cadmium | 64.23 | 25.27 |
| Sulfur | 22.60 | 31.17 |
| Carbon | 7.31 | 26.92 |
| Oxygen | 4.73 | 13.08 |
| Nitrogen | 1.13 | 3.56 |
|  | 100.00 | 100.00 |


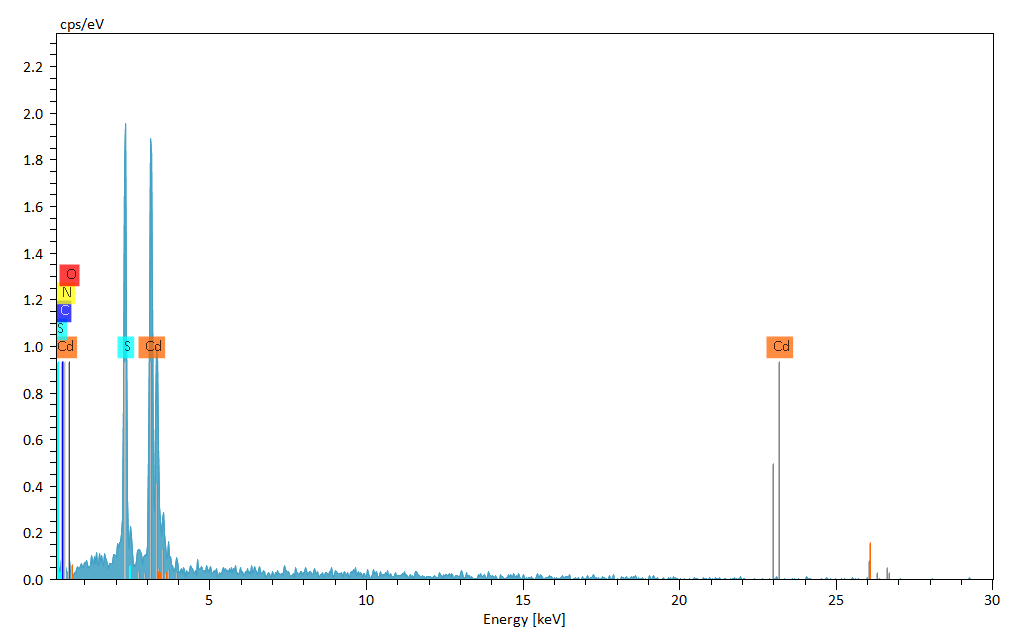


**Supplementary figure 2**. EDX spectrum of CQDs-SH/CdS QDs


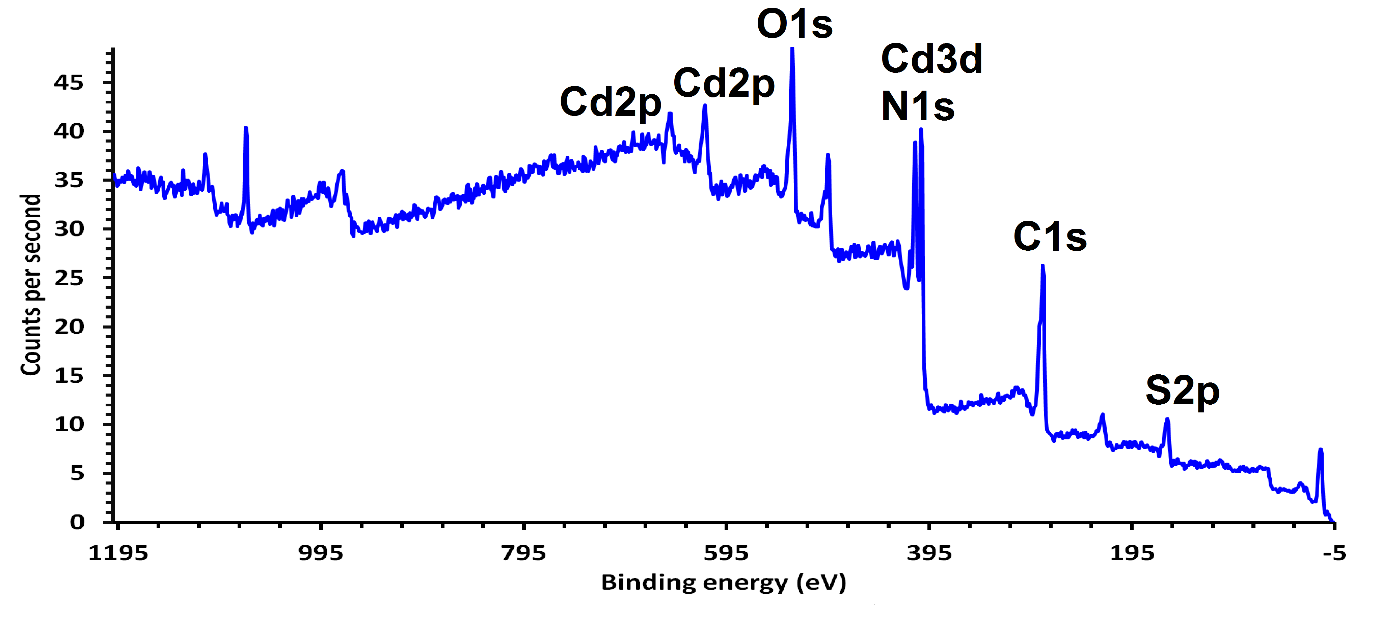


**Supplementary figure 3.**  XPS spectral of CQDs-SH/CdS QDs


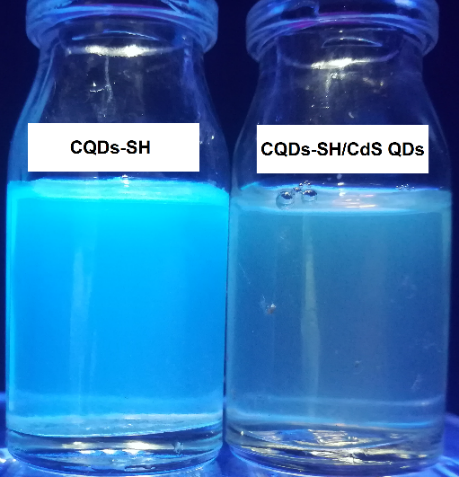


**Supplementary figure 4.** Photographs of an aqueous dispersion of CQDs-SH and CQDs-SH/CdS QDs under 365 nm UV irradiation


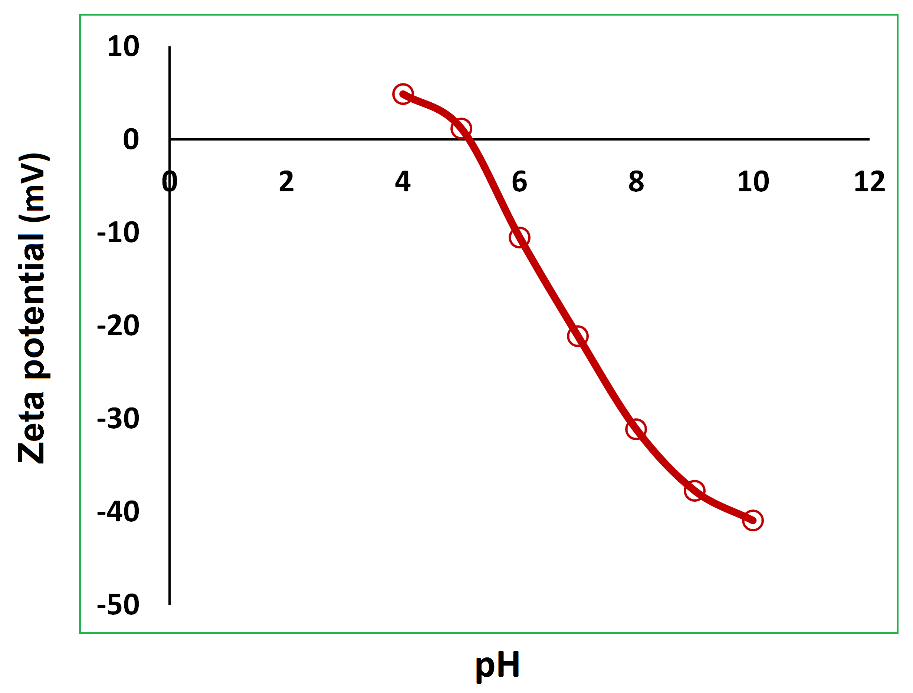


**Supplementary figure 5.** The zeta potential study of CQDs-SH/CdS QDs at different pH values

**
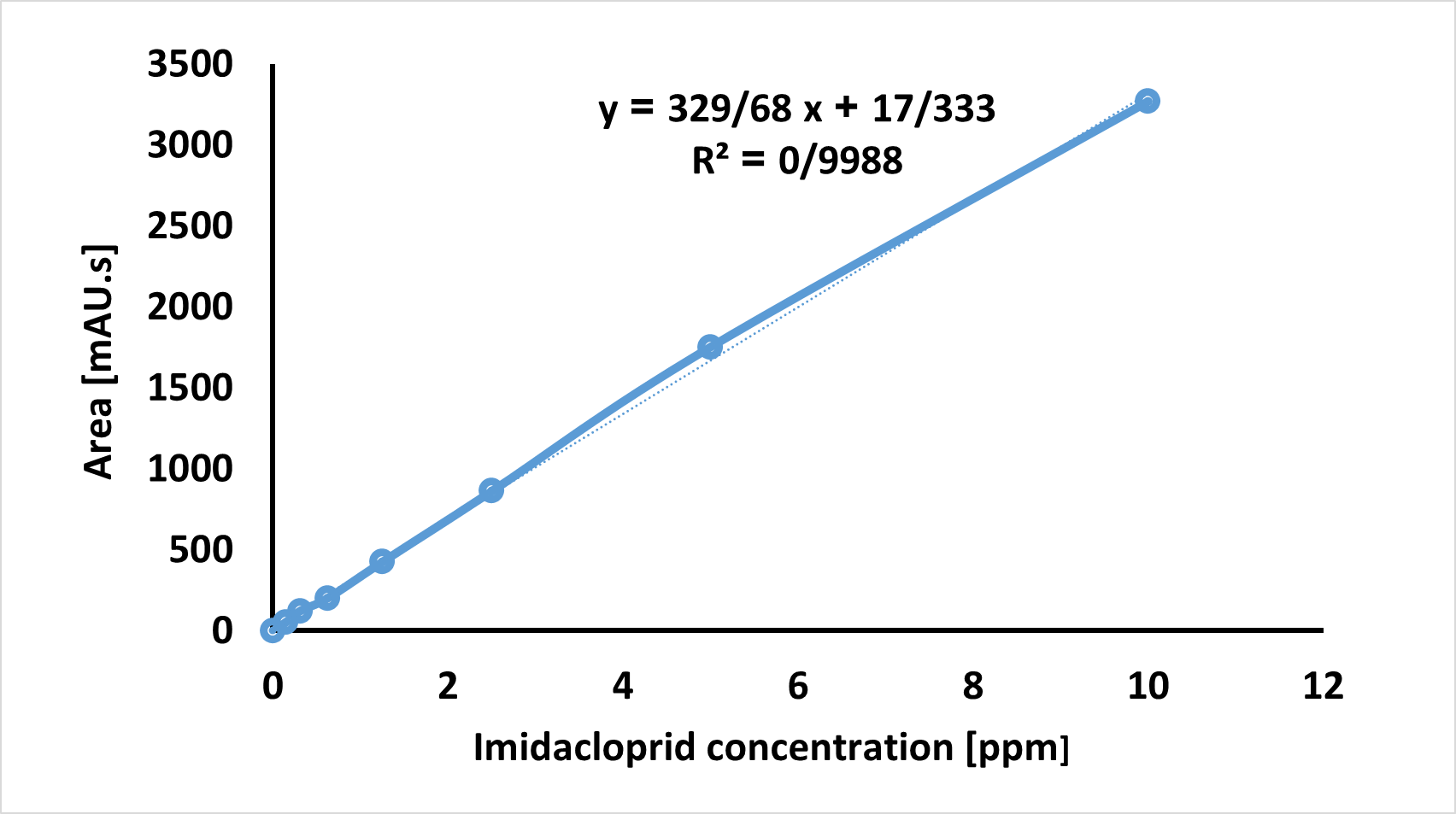
**

**Supplementary figure 6.** Calibration curve for HPLC


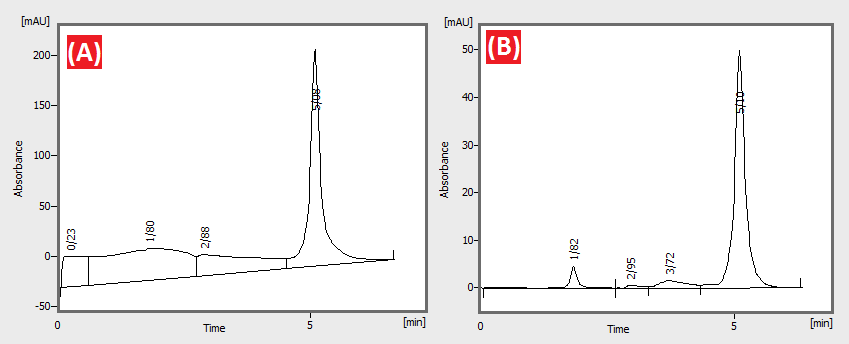


**Supplementary figure 7.** HPLC chromatogram of imidacloprid (10 mg/L) (a). HPLC chromatograms of imidacloprid solution after 90 min irradiation in the presence of CQDs-SH/CdS QDs, Reaction conditions: under simulated visible light, pH = 7, [imidacloprid] = 10 ppm, at 25 °C and in the presence of 1 g/L of photocatalyst.

**
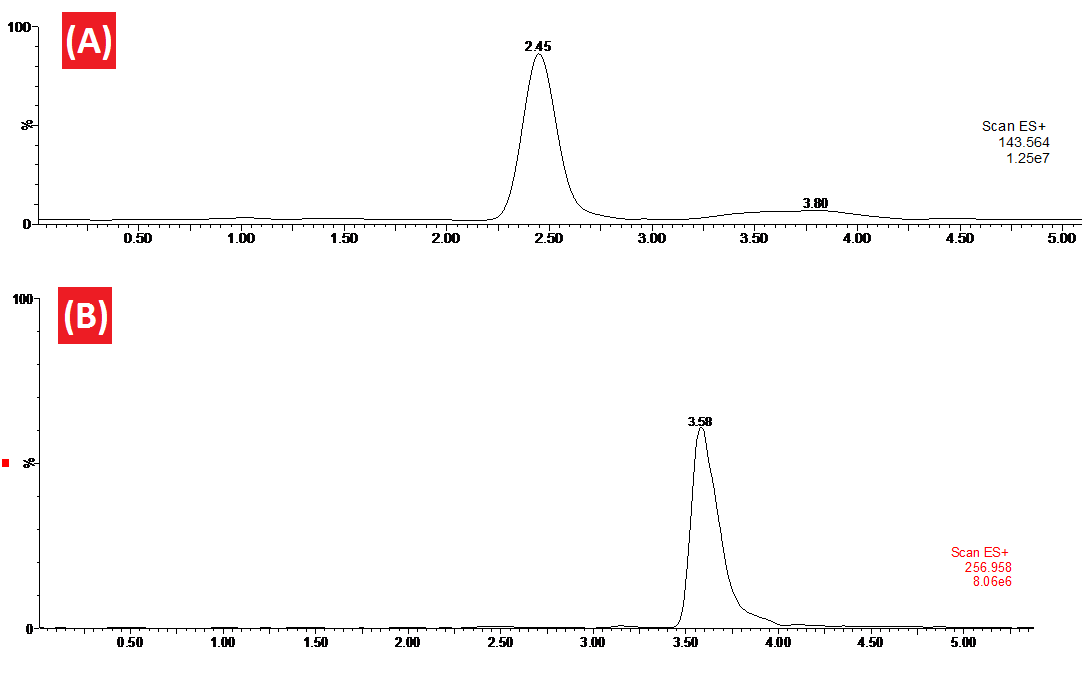
**

**Supplementary figure 8.** LC-MS chromatograms of the degradation product of imidacloprid at (A) (m/z=142) and (B) (m/z=257).

**
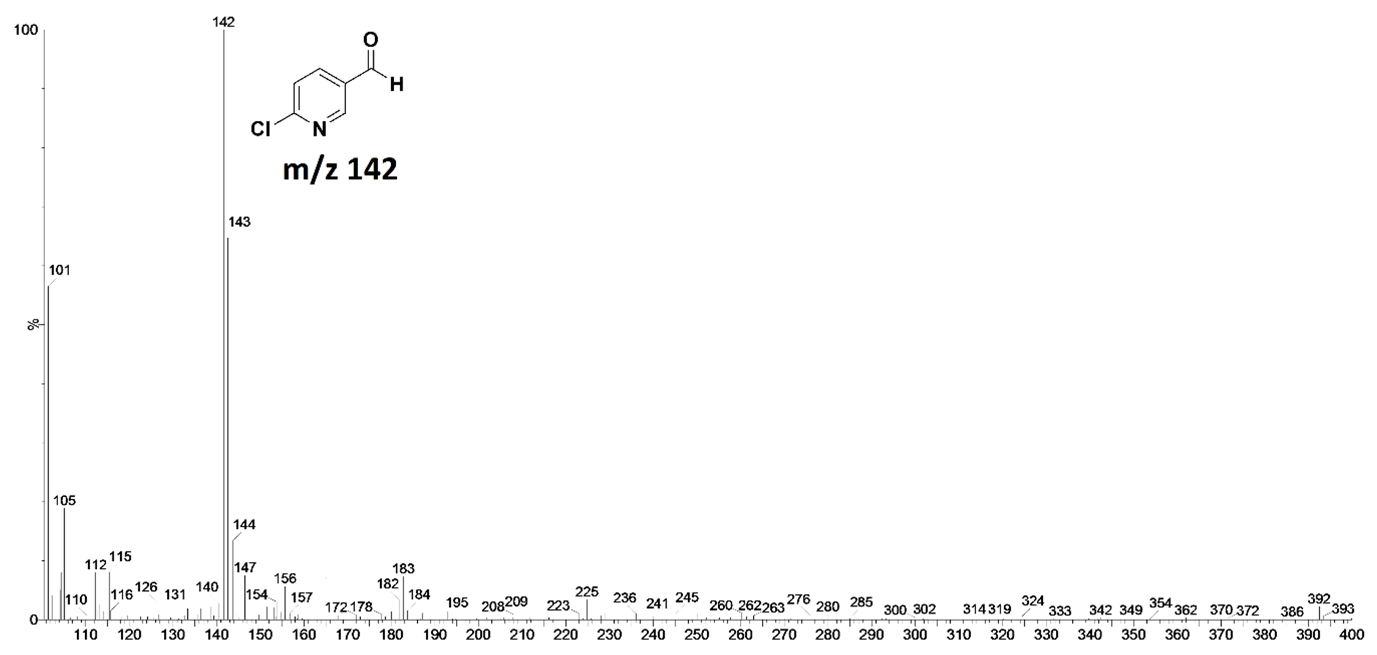
**

**Supplementary figure 9.** Mass spectrum the degradation product (m/z=142).

**
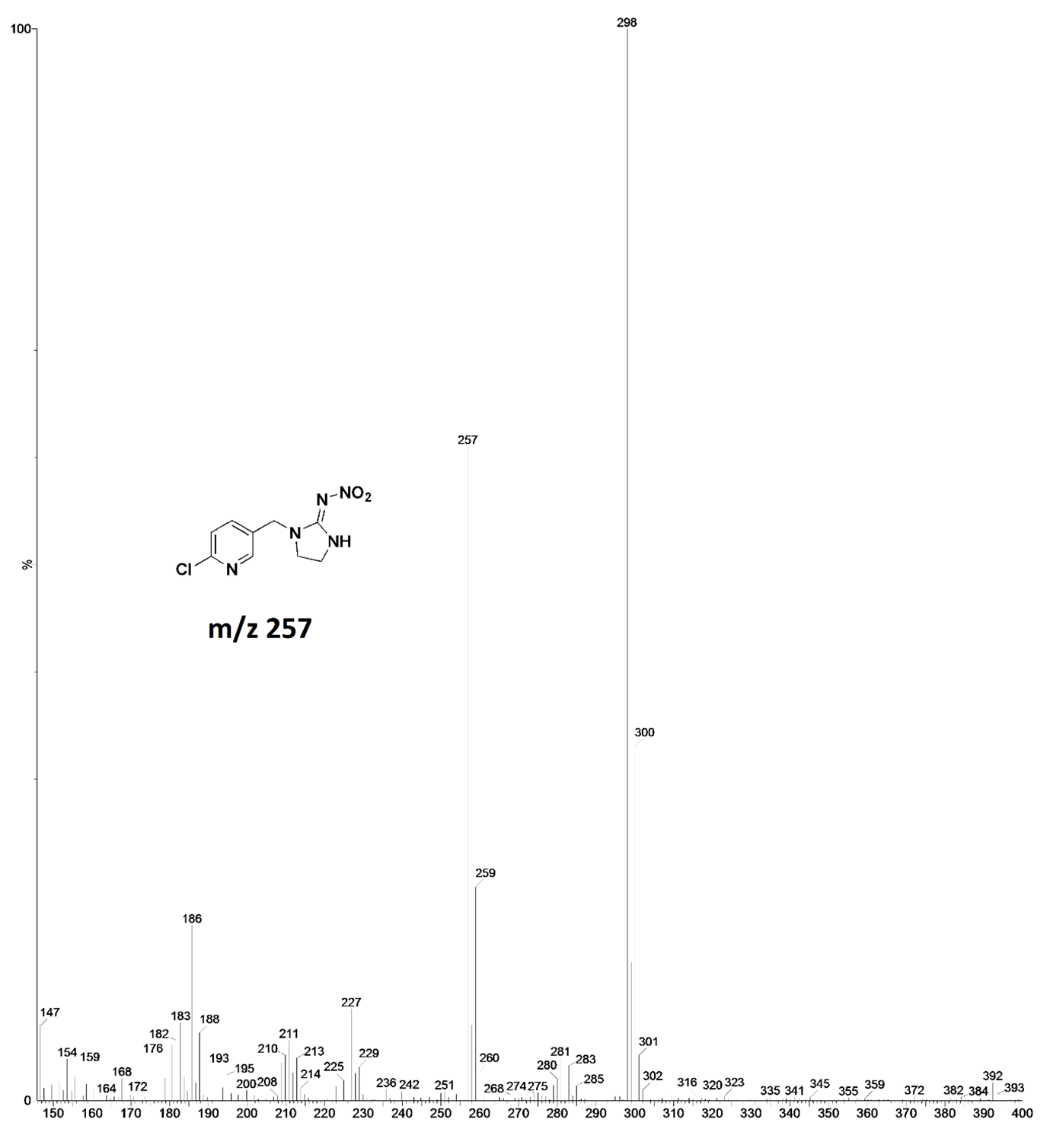
**

**Supplementary figure 10.** Mass spectrum of imidacloprid (m/z=257).
